# Factors that Affect the Osteoclastogenesis of RAW264.7 Cells

**DOI:** 10.16966/2576-5833.109

**Published:** 2017-08-28

**Authors:** John Nguyen, Anja Nohe

**Affiliations:** Department of Biological Sciences, University of Delaware, Newark, USA

**Keywords:** RAW264.7, Osteoclastogenesis, Osteoclast Culture Protocol, Macrophage Polykaryons

## Abstract

Osteoclasts and their activity are key regulators of bone formation. However, studying osteoclasts is difficult. Primary osteoclast cultures are difficult to maintain and isolate. Also, the amount of cells that are isolated and their properties depend on the origin and differentiation protocols. These protocols are usually developed in a distinct lab and multiple protocols exist. A cell line to study osteoclasts and a thorough study of osteoclast differentiation and culturing is currently lacking. The RAW264.7 cell line is most commonly used to study osteoclast differentiation and its signaling pathways. RAW264.7 cells are not a homogenous cell line. They don’t often exclusively differentiate into osteoclast but also into other multinucleated cells as well including macrophage polykaryons. A challenge of culturing RAW264.7 cells are culture conditions. Different conditions can affect survival, proliferation, and differentiation of RAW264.7 cells. Currently published protocols of culturing RAW264.7 cells often assume multinucleated cells that have three or more nuclei with distinguished osteoclast characteristics (such as TRAP+) as osteoclasts. However, osteoclasts and macrophage polykaryons are almost indistinguishable under a light microscope (TRAP+ with three or more nuclei). The goal of this paper is to examine the effect of culture conditions on the osteoclastogenesis ability of RAW264.7 cells. The focus will be on establishing the crucial parameters for culture density, time of stimulation, RANKL, and L-Gln concentrations. Although we are unable to establish the condition that offers a homogenous population of osteoclasts; nevertheless, we are able to identify the optimal conditions at which osteoclasts are found to be more than macrophage polykaryons. Finally, this article also demonstrates that osteoclasts and macrophage polykaryons can be distinguished by immunofluorescence staining for cathepsin K.

## Introduction

Osteoclasts are multinucleated cells that are essential for bone resorption and regulate bone remodeling. Dysregulation of osteoclasts can cause bone diseases such as osteopetrosis and osteoporosis. Osteoclasts can be obtained by isolating primary bone marrow monocytes or by using macrophage cell lines. In both cases, the cells need to be differentiated into the mature osteoclast by M-CSF and RANKL [1–4]. Disadvantages of primary cells include the difficulty of obtaining a true homogenous population, sensitivity, and requirement of additional nutrients. The murine macrophage cell line RAW264.7 was first established almost four decades ago [5,6]. Since then it has become an important cell line to study monocyte differentiation. Recently, this cell line has become a valuable tool to study osteoclast differentiation and activity due to its expression of RANK and differentiation to osteoclast in response to RANKL [7]. Unlike with BMMs, RAW264.7 can secret M-CSF on its own, thus, co-culture with M-CSF is unnecessary [8].

However, RAW264.7 also isn’t a homogeneous cell line. It is fairly well-known that different laboratories have different populations that are more or less able to become multinucleated. Recent reports show that the culture conditions for these cells are extremely important. For example, research shows that cell density affects the stimulation of RAW264.7 cell line [9]. Moreover, contradictory results using these cell line have been reported [10,11]. Previously, there were several published protocols of the culture of RAW264.7 cell line [8,12,13]. Although these protocols provide a successful method to culture osteoclasts from RAW264.7 cells, they don’t express the issue of having multinucleated cells such as macrophages mixing with osteoclasts in the population. It has been shown that multinucleated foreign body giant cells also express tartrate-resistant acid phosphatase (TRAP) [14], hence, classical TRAP staining is unable to distinguish between macrophage polykaryons and osteoclasts. Furthermore, the importance of L-Gln to osteoclast culture has not been paid enough attention in these protocols. A study from Indo et al. [15] demonstrated the importance of L-Gln for osteoclast differentiation of murine BMMs. Nevertheless, byproducts of L-Gln breakdown could be potentially harmful to cells [16].

The goal of this paper is to examine the effect of different culture conditions on the osteoclastogenesis ability of RAW264.7 cells. The focus will be on establishing culture density, time of stimulation, and RANKL and L-Gln concentrations. Although we are unable to establish the condition that offers a homogenous population of osteoclasts; nevertheless, we are able to identify the optimal conditions at which osteoclasts are found to be more than macrophage polykaryons. Finally, this article will also demonstrate that osteoclasts and macrophage polykaryons can be distinguished by immunofluorescence.

## Material and Method

### Reagents

Fetal bovine serum (Gemini Bio-Product, Cat#100-106, Lot#A39E00F). Dulbecco’s Modification of Eagle’s Medium (DMEM with 4.5 g/L glucose without sodium bicarbonate, L-glutamine, and sodium pyruvate; Corning, Cat#90-013-PB, Lot#90013165). L-glutamine (L-Gln; Gemini Bio-Product, Cat#400-106, Lot#F11P00F). Penicillin/Streptomycin (Gemini Bio-Product, Cat#400-106, Lot#F24P00G). Sodium pyruvate (Corning, Cat#25-000Cl, Lot#34815011). RANKL (Sino Biological Inc., Cat#11682-HNCH-100). ELF97 alkaline phosphatase substrate (Life Technologies, Cat#E6588, Lot#1704566). Cathepsin K (Santa Cruz, Cat#6506, Lot#J613). Alexa fluor 568 (Invitrogen, Cat#A11057, Lot#622091). Acid phosphatase, leukocyte (TRAP) kit (Sigma-Aldrich 387-1KT).

### L-Gln concentration

RAW264.7 cells were seeded at 0.45 × 10^4^ cells/cm^2^ in a 6-well-plate in DMEM culture media (10%FBS v/v, 1%Pen/Strep v/v, 1% sodium pyruvate v/v, 4.5 g/L glucose, and 1.8 g/L sodium bicarbonate). After 2 hours, they were stimulated with RANKL 10ng/ml in DMEM culture media with 0, 0.5, 1, 2, 4 or 6mM L-Gln. The experiment was carried out for 5 days. Media was changed with fresh L-Gln and RANKL on day 3. Three independent trials were performed.

### RANKL concentration

RAW264.7 cells were seeded at 0.45 × 10^4^/cm^2^ in a 6-well-plate in DMEM culture media (10%FBS v/v, 1%Pen/Strep v/v, 1% sodium pyruvate v/v, 4.5 g/L glucose, 1.8 g/L sodium bicarbonate, and 2mM L-Gln). After 2 hours, they were non-stimulated or stimulated with RANKL 10ng/ml, 25ng/ml, 50ng/ml, 100ng/ml or 200ng/ml in DMEM culture media for 5 days. Media was changed with fresh RANKL on day 3. Three independent trials were performed.

### Cell density

RAW264.7 cells were seeded at different densities 0.225 × 10^4^ cells/cm^2^, 0.45 × 10^4^ cells/cm^2^, 0.9 × 10^4^ cells/cm^2^, 1.8 × 10^4^ cells/cm^2^, 3.6 × 10^4^ cells/cm^2^ or 7.2 × 10^4^ cells/cm^2^ in DMEM culture media (10%FBS v/v, 1%Pen/Strep v/v, 1% sodium pyruvate v/v, 4.5 g/L glucose, 1.8 g/L sodium bicarbonate, and 2mM L-Gln) in 6-well-plate. Cells were allowed to attach and grow for 2 hours, 1 day, 2 days, or 3 days before stimulated with RANKL 10ng/ml. The experiment was carried out for 5 days. Another set of plates were seeded as described above but were not treated with RANKL to serve as a control. Media was changed with fresh RANKL on day 3. Three independent trials were performed.

### TRAP staining

Media was removed and cells were washed with PBS thrice. They were fixed with 4% paraformaldehyde for 15 minutes at room temperature and washed for 5 minutes while shaking thrice. Then they were stained for tartrate-resistant acid phosphatase (Sigma-Aldrich) according to manufacturer protocol for 90 minutes at 37°C away from light. Nuclei were counterstained with Hematoxylin Gill No.3 for 2–3 minutes. Positive TRAP cells were visualized by a light microscope using 20X objective. Images were processed by ImageJ. The Same setting of color balance, brightness, and contrast was adjusted for each image. Traditionally, osteoclast was identified if it had three or more nuclei.

### ELF97 phosphatase substrate

RAW264.7 cells were seeded at 0.9 × 10^4^ cells/cm^2^ on coverslips plated in 30mm dishes. Cells were allowed to attach for 2 hours before stimulated with RANKL 10ng/ml for 5 days. Another set of coverslips were set up but weren’t stimulated with RANKL to serve as a control. Media was changed on day 3.

After 5 days, media was removed and cells were washed with PBS thrice. Then they were fixed with 4% paraformaldehyde for 15 minutes at room temperature and washed with PBS for 5 minutes while shaking thrice. Then, they were incubated in an ELF97 solution (110mM acetate buffer, pH5.2, 1.1 mM sodium nitrite, 7.4mM tartrate, 200μM ELF97 phosphatase substrate) for 1 min, 5 min, 10 min, 15 min, 30 min or 60 min at room temperature away from light. Coverslips were mounted onto slides with Airvol mounting medium. They were observed by using fluorescence microscope using 100X objective.

### Immunofluorescence

RAW264.7 cells were seeded at 0.9 × 10^4^ cells/cm^2^ on coverslips plated in 30mm dishes. Cells were allowed to attach for 2 hours before stimulated or non-stimulated with RANKL 10 ng/ml for 5 days. Media was changed on day 3.

After 5 days, media was removed and cells were washed with PBS thrice. Then they were fixed and permeabilized by submerging coverslips in ice-cold methanol for 10 min at −20°C followed by ice-cold acetone for 1 min at −20°C. Non-specific binding was blocked with 3% BSA for 1 hour at room temperature. Incubated with 1° antibody (goat polyclonal Cathepsin K (1:50 dilution in 1%PBS) for 1 hour at room temperature (secondary control was incubated with 3% BSA instead) followed by 2° antibodies (Alexa 568 donkey anti-goat (1:500 dilution in 1%PBS) for 1 hour protected from light. Nuclei were stained with Hoechst (1:10000 dilution in deionized water) for 1 min protected from light. Coverslips were mounted onto slides with Airvol mounting medium and observed with a fluorescence microscope using 100X objective.

### Statistics

Results were analyzed by one-way ANOVA and outliners were eliminated by Chauvenet’s criterion.

## Result

### Formation of TRAP-positive multinucleated by RAW264.7 cells in the absence of RANKL

**Figure 1:**
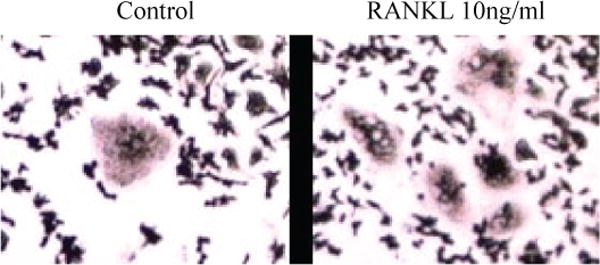
RAW264.7 cells form multinucleated cells in the absence of RANKL. Cells were cultured at a density of 1.8 × 10^4^ cells/cm^2^ and stimulated with or without RANKL 10ng/ml. Multinucleated osteoclasts were defined as having three or more nuclei.

In order to test whether RAW264.7 cells stain positive for TRAP in the absence of RANKL, we cultured RAW264.7 cells for 5 days. As can be seen, the cells differentiated into macrophages and macrophage polykaryons and they both stained positive for TRAP and multinucleated with three or more nuclei as described by [17,18]. In comparison, we stimulated the cells with 10ng/ml RANKL and observed an increase in the number of TRAP-positive multinuclear cells (Figure 1).

### RANKL promoted osteoclastogenesis of RAW264.7 cells at 10ng/ml, but higher concentration of RANKL didn’t enhance osteoclastogenesis further

**Figure 2:**
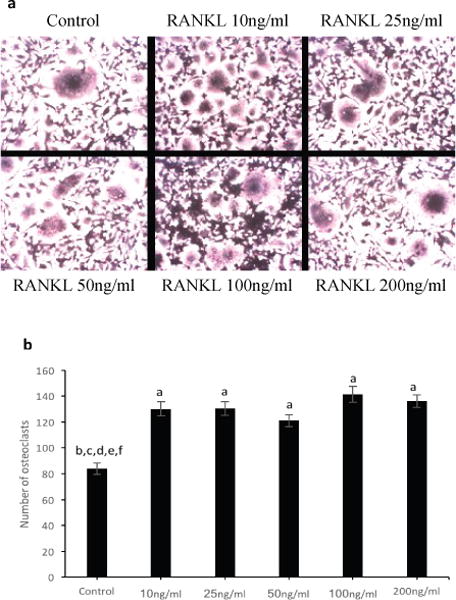
Effect of concentration of RANKL on osteoclastogenesis of RAW264.7 cells. RAW264.7 cells were cultured in DMEM culture media at 0.45 × 10^4^ cells/cm^2^ and were non-stimulated or stimulated with 10ng/ml, 25ng/ml, 50ng/ml, 100ng/ml, or 200ng/ml of RANKL for 5 days. Multinucleated cells were stained for TRAP. Multinucleated osteoclast was defined as having three or more nculei.a) Representative images of multinucleated cell formation mediated by RANKL at indicated concentration. b) Quantitative analysis of amount of TRAP positive multinucleated cells. Statistical significant (P<0.05) was analyzed by one-way ANOVA.

To determine the optimal concentration of RANKL for the differentiation of RAW264.7 cells to osteoclasts, RANKL concentration curve was performed (Figure 2a). RAW264.7 cells were cultured in DMEM culture media at 0.45 × 10^4^ cells/cm^2^ (10%FBS v/v, 1%Pen/Strep v/v, 1% sodium pyruvate v/v, 4.5 g/L glucose, 1.8 g/L sodium bicarbonate, and 2mM L-Gln) and were non-stimulated or stimulated with 10ng/ml, 25ng/ml, 50ng/ml, 100ng/ml, or 200ng/ml of RANKL for 5 days. Media was exchanged on day 3 with new RANKL added. Multinucleated cells were stained for TRAP on day 5. Images per well were taken. TRAP-positive multinucleated cells were counted in each image. Data were normalized to non-RANKL stimulation control. Data are shown as mean ± SEM. Experiments were performed in triplicates. The appearance of TRAP-positive in control stimulation indicated the formation of multinucleated cells in a normal osteoclast culture system from a RAW264.7 cell line (Figure 2b). The result indicated that RANKL significantly promoted total multinucleated cell formation of RAW264.7 cells at 10ng/ml. However, RANKL didn’t enhance the total number any further at concentrations above 10ng/ml.

### L-Gln enhanced multinucleated cell differentiation of RAW264.7 cells at 1–2mM but inhibited at higher concentrations

**Figure 3:**
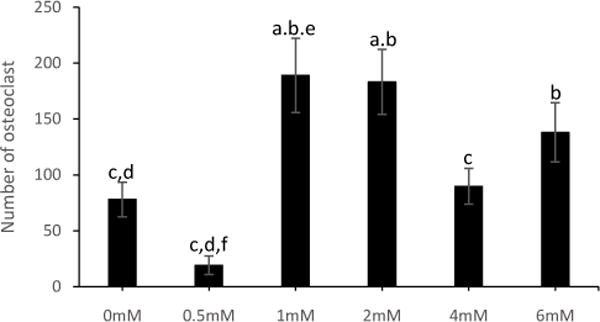
Effect of concentration of L-Gln on osteoclastogenesis of RAW264.7 cells. RAW264.7 cells were cultured in DMEM culture media with RANKL 10ng/ml for 5 days at 0.45 × 10^4^ cells/cm^2^. Cells were stained for TRAP.

L-Gln is important for cell growth and proliferation. However, byproducts of L-Gln, ammonia/ammonium ions, are potentially harmful and have negative effects on growth and proliferation. Therefore, we determined the optimal concentration of L-Gln for the culturing system and tested various L-Gln concentrations (Figure 3). RAW264.7 cells were cultured in DMEM culture media (10%FBS v/v, 1%Pen/Strep v/v, 1% sodium pyruvate v/v, 4.5 g/L glucose, 1.8 g/L sodium bicarbonate, and 0mM, 0.5mM, 1mM, 2mM, 4mM or 6mM L-Gln) with RANKL 10ng/ml for 5 days at 0.45 × 10^4^ cells/cm^2^. New media was exchanged on day 3 and new RANKL added. Multinucleated cells were stained for TRAP on day 5. Images per well were taken. TRAP-positive multinucleated cells were counted in each image. Data were normalized to 0mM L-Gln control. Data are shown as mean ± SEM. Experiments were performed in triplicates. Statistical significant (P<0.05) was analyzed by one-way ANOVA. The result showed that L-Gln enhanced multinucleated cell differentiation of RAW264.7 cells at 1–2mM significantly. However, as concentration increased to 4mM the differentiation was inhibited. At 6mM there was no statistical difference between 1–2mM or control, however, it showed a decreasing trend. Noteworthy, even though there was no statistical significance between 4mM and 6mM L-Gln, however, there was an upward trend from 4mM to 6mM. Thus, it could be speculated that higher concentration of L-Gln could increase osteoclast differentiation again and even exceed the effect of 1mM or 2mM L-Gln. Future investigation should be carried out to further determine the effect of higher concentration of L-Gln on osteoclast differentiation of RAW264.7 cells.

### Osteoclast differentiation of RAW264.7 cells was dependent on seeding density

**Figure 4:**
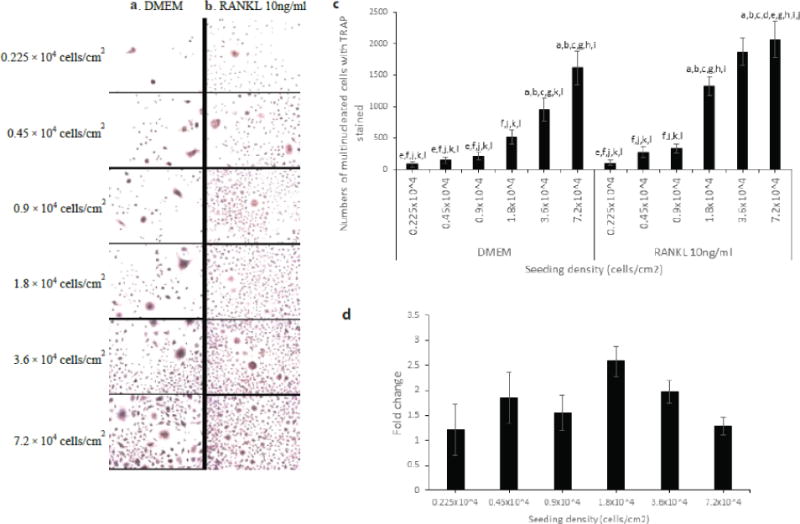
Effect of seeding density on osteoclast differentiation of RAW264.7 cells. RAW264.7 cells were seeded at 0.225 × 10^4^ cells/cm^2^, 0.45 × 10^4^ cells/cm^2^, 0.9 × 10^4^ cells/cm^2^, 1.8 × 10^4^ cells/cm^2^, 3.6 × 10^4^ cells/cm^2^ or 7.2 × 10^4^ cells/cm^2^ and cultured in DMEM culture media. After 2 hours, they were non-stimulated or stimulated with RANKL 10ng/ml for 5 days. Multinucleated cells were stained for TRAP. Osteoclast was defined as having three or more nculei. **a)** Representative images of multinucleated cell formation at different seeding density in non-RANKL stimulated**. b)** Representative images of multinucleated cell formation at different seeding density in RANKL stimulated **c)** Numbers of multinucleated cells that were TRAP positive**. d)** Fold change of multinucleated cells with TRAP stained between RANKL stimulation and DMEM control. Number of multinucleated cells of RANKL stimulation was divided by number of multinucleated cells of DMEM control atsame density.

Next, we evaluated the effect of seeding density on osteoclast differentiation. Seeding density is a major parameter for cell differentiation [19]. For example, in 1000–2000 cells/cm^2^ it was shown that cell density enhanced the differentiation but decreased at 4000–8000 cells/cm^2^. To evaluate the effect of seeding density on a number of osteoclasts vs multinucleated cells, RAW264.7 cells were seeded at different densities (Figure 4a and 4b). RAW264.7 cells were seeded at 0.225 × 10^4^ cells/cm^2^, 0.45 × 10^4^ cells/cm^2^, 0.9 × 10^4^ cells/cm^2^, 1.8 × 10^4^ cells/cm^2^, 3.6 × 10^4^ cells/cm^2^ or 7.2 × 10^4^ cells/cm^2^ and cultured in DMEM culture media (10%FBS v/v, 1%Pen/Strep v/v, 1% sodium pyruvate v/v, 4.5 g/L glucose, 1.8 g/L sodium bicarbonate, and 2mM L-Gln). After 2 hours, they were non- stimulated or stimulated with RANKL 10ng/ml for 5 days. On day 3 media was exchanged and new RANKL added Multinucleated cells were stained for TRAP. Images per well were taken. TRAP-positive multinucleated cells were counted in each image (Figure 4c). Fold change was calculated by dividing a number of multinucleated cells of RANKL stimulation to a number of multinucleated cells of DMEM control of same density (Figure 4d). Fold change was used to estimated the amount of osteoclasts were generated at each different seeding density. Data are shown as mean +/−SEM. Experiments were performed in triplicates. Statistical significant (P<0.05) was analyzed by one-way ANOVA. Multinucleated cell formation increased as seeding density increased (Figure 4c) in both nonstimulated and stimulated RANKL 10ng/ml. In addition, it showed that seeding density of 1.8 × 10^4^ yielded a higher amount of osteoclasts over multinucleated cells (Figure 4d).

### Delayed starting time of stimulation by 24 hours enhanced osteoclast differentiation of RAW264.7 cells

**Figure 5:**
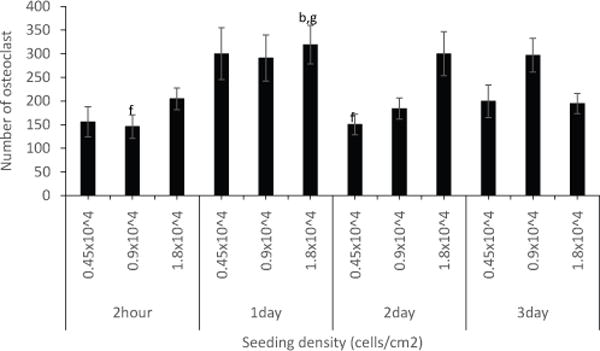
Effect of time of stimulation on osteoclast differentiation of RAW264.7 cells. RAW264.7 cells were seeded at 0.45 × 10^4^ cells/cm^2^, 0.9 × 10^4^ cells/cm^2^, or 1.8 × 10^4^ cells/cm^2^ and cultured in DMEM culture media. They were allowed to adhere for 2 hours, 1 day, 2 days, or 3 days before stimulated with RANKL 10ng/ml or DMEM for 5 days. Multinucleated cells were stained for TRAP. Osteoclast was defined as having three or more nculei.

Different research groups often use different protocols for RAW264.7 culture. Each often indicates different starting time of stimulation. For example, one group would prefer to start stimulation immediately after plating, while another would wait after an overnight incubation [11,12]. To determine whether or not osteoclast formation of RAW264.7 cells was dependent on the starting time of stimulation, RAW264.7 cells were seeded and stimulated with RANKL after 2 hours, 1 day, 2 days, or 3 days (Figure 5). RAW264.7 cells were seeded at 0.45 × 10^4^ cells/cm^2^, 0.9 × 10^4^ cells/cm^2^, or 1.8 × 10^4^ cells/cm^2^ and cultured in DMEM culture media (10%FBS v/v, 1%Pen/Strep v/v, 1% sodium pyruvate v/v, 4.5 g/L glucose, 1.8 g/L sodium bicarbonate, and 2mM L-Gln). They were allowed to adhere for 2 hours, 1 day, 2 days, or 3 days before stimulated with RANKL 10ng/ml or DMEM for 5 days. After 3 days following stimulation, media was replaced and cells stimulated with new RANKL. At day 5 cells were fixed and stained. Images per well were taken. TRAP-positive multinucleated cells were counted in each image. The number of osteoclasts was estimated by dividing total multinucleated cells from RANKL stimulation to its corresponded DMEM control. Data are shown as mean ± SEM. The result indicated that stimulation of cells after 1 day of seeding showed an increase in osteoclast number at all three tested densities over control 2-hour- delayed-stimulation. Densities of 1.8 × 10^4^ cells/cm^2^ and 0.9 × 10^4^ cells/cm^2^ at 2-day-delayed-stimulation and 3-day-delayed-stimulation, respectively, showed similar increase with 1-day-delayed-stimulation.

### Multinucleated cells and osteoclasts couldn’t be distinguished by ELF97 alkaline phosphatase substrate

**Figure 6:**
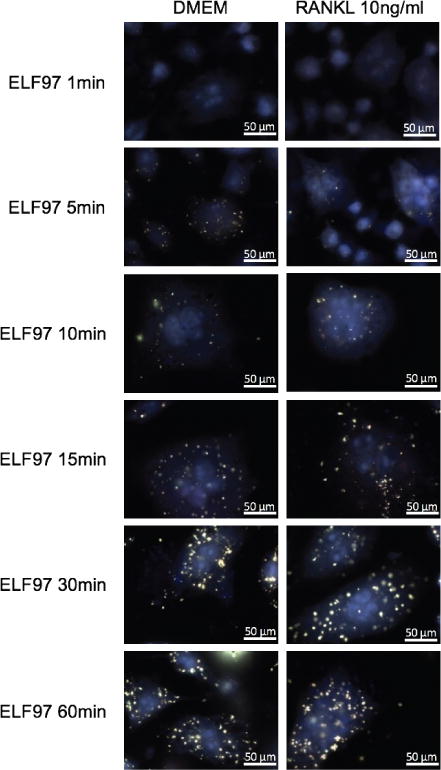
Fluorescence-based TRAP staining by ELF97 alkaline phosphatase substrate. Cells were seeded at 0.9 × 10^4^ cell/cm^2^ and grew on coverslips. Stimulated with RANKL 10ng/ml or with DMEM for 5 days. Fixed with 4% paraformaldehyde and incubate in ELF97 solution for 1min, 5min, 10min, 15min, 30min, or 60min.

ELF97 phosphatase substrate is an enzyme-labeled fluorescence substrate. It is used and proved to be specific to detect osteoclasts [20]. Therefore, to distinguish between multinucleated cells and osteoclasts, fluorescence-base TRAP by ELF97 phosphatase substrate was employed (Figure 6). Cells were seeded at 0.9 × 10^4^ cell/cm^2^ and grew on coverslips. Stimulated with RANKL 10ng/ml or with DMEM for 5 days. Fixed with 4% paraformaldehyde and incubate in ELF97 solution (110mM acetate buffer, pH5.2, 1.1 mM sodium nitrite, 7.4mM tartrate, 200μM ELF97 phosphatase substrate) for 1min, 5min, 10min, 15min, 30min, or 60min. Cells were visualized with a fluorescence microscope using oil 100X objective. Nevertheless, fluorescence was also detected in control DMEM stimulation. It wasn’t a surprise since it had been reported that multinucleated cells also expressed tartrate-resistant acid phosphatase.

### Multinucleated cells and osteoclasts can be distinguished by immunofluorescent staining for Cathepsin K

**Figure 7:**
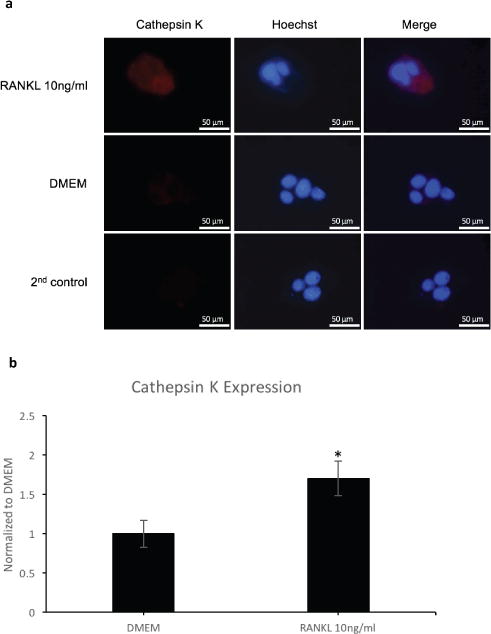
Immunofluorescence of multinucleated cells and osteoclasts. Cells were seeded at 0.9 × 10^4^ cells/cm^2^ and grew on coverslips. Stimulated with RANKL 10ng/ml or with DMEM for 5 days. Fixed and permeabilized in methanol at −20°C followed by acetone at −20°C. Blocked with 3%BSA. Incubated with goat polyclonal Cathepsin K followed by Alexa 568 donkey anti-goat. Nuclei was stained with Hoechst. **a)** Representative images **b)** Quantitative analysis of Cathepsin K expression.

Because ELF97 phosphatase failed to distinguish between multinucleated cells and osteoclasts, we turned to immunofluorescence using an antibody against Cathepsin K, a marker for osteoclast. Cells were seeded at 0.9 × 10^4^ cells/cm^2^ and grew on coverslips. Stimulated with RANKL 10ng/ml or with DMEM for 5 days. Fixed and permeabilized in methanol at −20°C followed by acetone at −20°C. Blocked with 3%BSA. Incubated with goat polyclonal Cathepsin K followed by Alexa 568 donkey anti-goat. Nuclei were stained with Hoechst. Cells were visualized with a fluorescence microscope using oil 100X objective. The result showed that only multinucleated cells stimulated with RANKL secreted Cathepsin K (Figure 7a). Quantitative analysis indicated higher expression of cathepsin K in RANKL-stimulated than in control (Figure 7b). Thus, it demonstrated that immunofluorescence could be used to distinguish between multinucleated cells and osteoclasts.

### BMP2 enhanced osteoclastogenesis of RAW264.7 under established culture conditions

**Figure 8:**
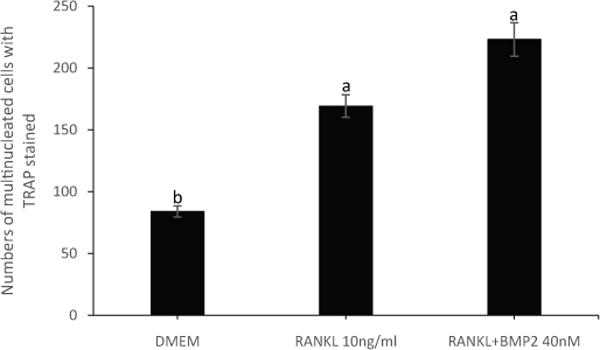
BMP2 enhanced osteoclastogenesis of RAW264.7 cells. Cells were seeded at 0.45 × 10^4^ cells/cm^2^ in a 6-well-plate. Stimulated for 5 days with RANKL 10ng/ml, RANKL+BMP2 40nM, or DMEM. Cells were fixed and stained for TRAP. Osteoclast was defined as having three or more nculei.

To confirm and validate the new established culture condition, cells were stimulated with BMP2. It was reported that BMP2 played a positive role in enhancing osteoclastogenesis of murine osteoclast precursors and the inhibition of BMP2 signaling via BMPRII knockdown suppressed osteoclastogenesis of RAW264.7 cells [21]. Nevertheless, BMPRII ligands included BMP2, BMP4, BMP6, BMP7, BMP10-14 [22]. Zheng et al. [23] reported that BMP2 homodimer increased osteoclastogenesis of RAW264.7 cells. The results showed that the addition of 40nM BMP2 to RANKL+ culture media enhanced osteoclast differentiation (Figure 8), thus, validating the established culture conditions.

## Discussion

Culturing of RAW264.7 cells has been employed for more than three decades for osteoclast and bone mineralization research. However, RAW264.7 cells are not homogeneous cell line. It is fairly known that different laboratories would have a different population that more or less able to become multinucleated. Hence, we purchased our RAW264.7 cell directly from American Type Culture Collection (ATCC) rather than obtained it from other laboratories. After undergoing 20 passages from the time received from ATCC, it was suggested by several published protocols that new cells should be used [8,12,13]. In addition, these protocols provide a good guideline on how to differentiate RAW264.7 cells to osteoclasts. For instance, these protocols showed that DMEM was also an effective differentiation media if co-culture with RANKL when it was used for RAW264.7 culture. Thus, DMEM was chosen to use in this study rather than alpha-DMEM. Nevertheless, they have not pressed the issue of obtaining multinucleated cells other than osteoclasts in the population. It was observed in this study that there was the formation of bigger cells (some with more than 3 nuclei) than monocyte in RAW264.7 passages before they were split and plated in a 6-well-plate for the experiment (data not shown). The formation of these bigger cells was observed even in passage lower than 20. These multinucleated cells also express TRAP [14]. Here, we showed that in our culture system multinucleated cells were formed in the absence of RANKL and were TRAP-positive (Figure 1). Thus, we were aiming to re-establish osteoclast culture method from RAW264.7 cells to combat this issue.

RANKL is a signaling factor of differentiation of osteoclasts. However, researchers have been using a different amount of RANKL for their culture systems depend on cell types. Here, we showed that RANKL effectively promoted total multinucleated cell formation at 10ng/ml. At higher concentration, RANKL didn’t increase this total number any further (Figure 2b). This suggested that formation of osteoclast and/or other multinucleated cells from RAW264.7 cells didn’t depend on the concentration of RANKL.

The importance of L-Gln in osteoclast culture medium was demonstrated by Indo et al. In that study, supplementation of L-Gln into L-Gln free media increased osteoclast differentiation of BMMs. The study showed L-Gln increased osteoclast differentiation from 0.5mM to 1.5mM.

However, the study didn’t show the effect of higher concentration of L-Gln on osteoclast differentiation.

L-Gln for decades has been recognized as an important factor for growth and differentiation of cells in culture systems [24,25]. The importance of L-Gln in osteoclast culture medium was demonstrated by Indo et al.[15]. In that study, supplementation of L-Gln into L-Gln free media increased osteoclast differentiation of murine BMMs. The study showed L-Gln increased osteoclast differentiation from 0.5mM to 1.5mM. However, the study didn’t show the effect of higher concentration of L-Gln on osteoclast differentiation. By-products of breaking down of L-Gln including ammonia/ammonium ions are toxic and harmful to cells [16]. Here, we showed that highest total multinucleated cells were formed at 1–2mM of L-Gln, but at 4–6mM of L-Gln the total of multinucleated cells was decreased (Figure 3b). This might be due to the accumulation of ammonia/ammonium ion from the breaking down of L-Gln in the culture system. Not surprisingly, a total number of multinucleated cells was lowest at 0–1mM of L-Gln.

Contact-inhibition via gap junctional communication between adjacent cells suppresses cell proliferation. Thus, it should come to no surprise that seeding density influent growth and proliferation in a culture system. But here, we showed that seeding density also influent the formation of osteoclasts as well as multinucleated cells. We observed that seeding density had a positive effect on the formation of total multinucleated cells (Figure 4c) whether or not RANKL was added to the culture media. We determined that formation of osteoclast was a peak at 1.8 cells/cm^2^ (Figure 4d). Taken together, these suggested that there was a positive correlation between seeding density and formation of multinucleated cells, but the formation of osteoclasts followed a normal distribution curve.

Next, we wanted to see if delayed stimulation would affect osteoclast differentiation of RAW264.7 cells. To our surprise, we found that stimulation after 1 day of seeding increased the amount of osteoclasts over multinucleated cells at all three tested densities (Figure. 5).

Next, we wanted to establish a method to distinguish between multinucleated cells and osteoclasts. For this, we performed fluorescence-based TRAP staining using ELF97 alkaline phosphatase substrate [20]. This phosphatase substrate was showed as a new method for detection of TRAP and provided better resolution [20]. Thus, it was employed in this study to distinguish between osteoclasts and macrophage polykaryons. ELF97, however, could be the substrate for other phosphatases. In order to lessen non-specific signaling, only cells with more than three nuclei were taken into account. However, the result showed that this method was not specific to only osteoclasts (Figure 6). It was observed that the dye would act as a substrate for all of the TRAP-secreted multinucleated cells including osteoclast and macrophage polykaryons. Hence, this made us turn to immunofluorescence method. Using antibody against Cathepsin K, a marker for osteoclast, we were able to distinguish between multinucleated cells and osteoclasts (Figure 7). Quantitative analysis of cathepsin K expression showed higher expression of cathepsin K in RANKL-stimulated cells. While cathepsin K is known to play a role in the degeneration of bone matrix protein such as type I and type II collagen [26], it is also identified to function in bone resorption of osteoclast [27,28]. In this study, bone resorption assay wasn’t performed to further investigate the differences between osteoclasts and other multinucleated cells. Nevertheless, the increase in bone resorptive function of osteoclast was suggested via the increase in cathepsin K expression (Figure 7). Another reason was that macrophage polykayons were reported to have bone resorptive ability like osteoclasts [29,30]. Thus, bone resorption assay would be excessive for the purpose of this paper.

## Conclusion

We were able to establish and optimize culture conditions of RAW264.7 cells for osteoclast culture. In addition, we didn’t find any evidence that different culture conditions affected osteoclast size (data not shown). Optimal culture conditions were confirmed by applying them to stimulation of RAW264.7 cells with BMP2 (Figure 8). The result showed BMP2 promoted differentiation of RAW264.7 cells to osteoclasts, which was similar what reported by Jensen and Zheng Y et al. [21,23]. Although, other multinucleated cells still was able to form in our culture system, however, we were able to establish a method to produce more osteoclasts than multinucleated cells.

## Abbreviations

M-CSF: Macrophage Colony-Stimulating Factor
RANKL: Receptor Activator of Nuclear Factor kappa-B Ligand
TRAP: Tartrate-Resistant Acid Phosphatase
SEM: Standard Error of the Mean
